# Transplant renal artery stenosis caused by the stretch of an artey branch: a case report and literature review

**DOI:** 10.1186/s12882-018-0856-y

**Published:** 2018-03-09

**Authors:** Xiaohang Li, Jialin Zhang, Yiman Meng, Lei Yang, Fengshan Wang, Baifeng Li, Xitong Zhang

**Affiliations:** 1 Department of Hepatobiliary Surgery and Organ Transplant, First Affiliated Hospital, China Medical University, No.155, Nanjing North Street, Shenyang, Liaoning Province 110001 People’s Republic of China; 2 Department of Intervention, First Affiliated Hospital, China Medical University, No.155, Nanjing North Street, Shenyang, Liaoning Province 110001 People’s Republic of China

**Keywords:** Transplant renal artery stenosis, Renal transplantation, Transluminal treatment, Stent implantation

## Abstract

**Background:**

Renal transplant is the preferred treatment option for these patients with end-stage renal disease. Transplant renal artery stenosis (TRAS) is one of the most common and serious vascular complications after renal transplantation, and most of the TRAS occurred in the anastomosis. The complication must be diagnosed and treated timely, otherwise the function of transplanted kidney may be losed.

**Case presentation:**

A 46-year-old male with end-stage renal disease of unknown cause received a cadaveric renal transplant one year ago. Although three antihypertensive medications were administrated, his blood pressure gradually increased to 190/120 mmHg 3 weeks posttransplantation. Also the level of creatinine increased to 194 μmol/L.Color Doppler ultrasonography indicated a decreased resistance index (RI) in intrarenal arteries and increased blood flow of the transplant renal artery, therefore, a vascular complication of TRAS was suspected. Arteriography was performed and demonstrated TRAS caused by stretch of an artery branch, and the TRAS occurred in the distal site of the anastomosis instead of the anastomosis. Percutaneous transluminal bare stent implantation treatment was successfully performed. Satisfactory clinical efficacy with improvement in transplant renal function and renovascular hypertension was achieved after the interventional treatment.

**Conclusion:**

To our knowledge this is the first reported case of TRAS caused by stretch of an artery branch. When refractory hypertension and allograft dysfunction are presented posttransplantation, TRAS should be suspected. Color Doppler ultrasonography as a non-invasive examination may provide some valuable information, three-dimention CT can be useful for further diagnosis, but is seldom necessary. Arteriography provides the definitive diagnosis of TRAS. Percutaneous transluminal stent implantation treatment of TRAS has high success rate with minimal invasion and complications. When an artery branch situated on the stenosis, a bare stent rather than covered stent is the preferred choice.

## Background

Compared to hemodialysis, renal transplantation provides superior long-term survival, high cost-effectiveness and improved living quality with patients with end stage renal disease. Therefore, most of these patients would choose renal transplantation as the primary treatment. There is a remarkable decrease in the rate of surgical complications, this may attribute to significant improvement and increased experience in surgical techniques. However, some serious vascular complications may cause the allograft lose. So these complications should be paid more importance. Transplant renal artery stenosis (TRAS), with an incidence of 1% to 23%, is an increasingly recognized complication of renal transplantation [1], which constitutes approximately 75% of vascular complications. TRAS could be diagnosed if a greater than 50% narrowing in the intraluminal diameter or a greater than 15 mmHg pressure drop crossing the stenotic segment were achieved [1, 2]. The most frequent site of TRAS was the surgical anastomosis, we report here a case that TRAS situated just beyond the anastomosis was caused by the stretch of an artery branch, a very rare reason, and he was cured by percutaneous transluminal stent implantation.

## Case presentation

**Fig. 1:**
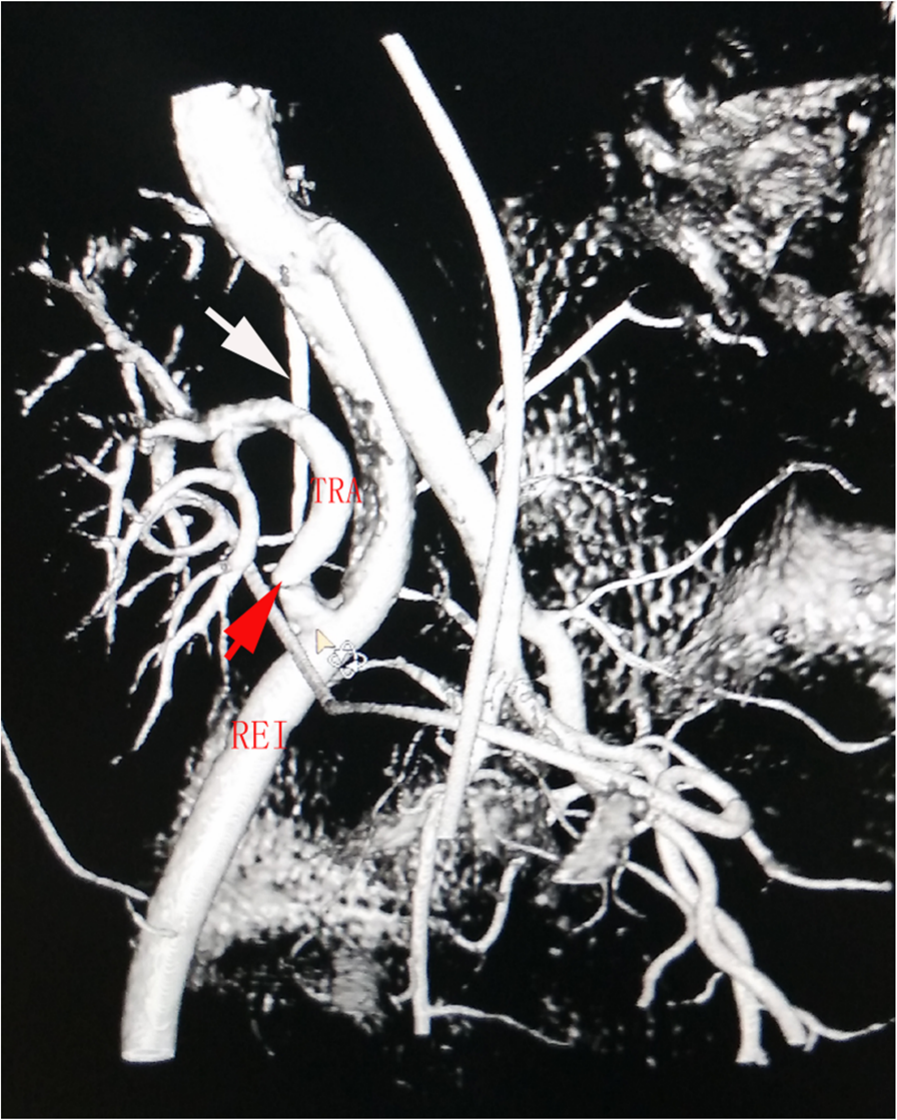
Digital subtraction angiography demonstrates a 90% stenosis of transplantation renal artery (red arrow). The white arrow shows the stretched renal artery branch. REI: right external iliac artery

A 46-year-old male with end-stage renal disease of unknown cause had been on hemodialysis for 3 years when he underwent cadaveric renal transplantation 15 months ago. Except for 3 years of hypertension, there was no other remarkable medical history. The kidneys of donor after brain death were procured by an operation group. However, during harvesting of the donor’s kidneys, a branch of right renal artery to renal superior polar was inadvertently cut off. The artery branch with a diameter 0.3 mm arised from the bifurcation of abdominal aorta and renal artery. Subsequently the severed renal artery branch was anastomosed in situ with 7–0 prolene during the repair of the kidney. Although the lengh of the renal artery branch became little shorter, the major trunk of renal artery was not excessively stretched. The cold and warm ischemia time of the kidney were 5 min and 6 h respectively. The right kidney of donor was transplanted in the right iliac fossa. The donor renal artery with a Carrel patch of donor aorta was anastomosed end-to-side to the recipient right external iliac artery with 6–0 prolene, and renal vein was anastomosed end-to-side to the recipient right external iliac vein with 5–0 prolene, and the graft ureter was anastomosed to the urinary bladder of the recipient with a double J stent. Basiliximab was used as immunity induction on the day of surgery and the fourth day after transplantation. His immunosuppression regimen consisted of tacrolimus, mycophenolate sodium, and prednisolone. His immediate postoperative course was unremarkable. His blood pressure was controlled to 140–156/90–105 mmHg with nicardipine spironolactone, and furosemide y. The 24-h urine volume was between 1937 and 4100 ml, and his renal function. Significantly improved, reaching a creatinine of 151 μmol/L on 11st day posttransplantation. However, from the 12nd day after transplantation his blood pressure began to gradually increase to 170–174/103-109 mmHg.There was no positive presentation on clinical examination and ultrasonograph, and the level of creatinine and urine volume were also stable. So the nicardipine was increased. To our surprise, it was still difficult to control his blood pressure even though three antihypertensive medications (sustained-release nifedipine 60 mg twice daily, urapidil 30 mg twice daily, arotinolol 10 mg twice daily) were administrated. His blood pressure reached to 190/120 mmHg on 20th day after transplantation. There was a decrease in 24-h urine volume with 1620-1725 ml, and mild impairment of renal function with a creatinine of 194 μmol/L. A bruit became audible over the site of the transplanted kidney. Color Doppler ultrasonography indicated a decreased RI in intrarenal arteries and increased blood flow of the transplant renal artery, with RI of 0.45, the peak systolic velocity(PSV) of 305 cm/s and the velocity gradient between stenotic and prestenotic segment of more than 3:1, therefore, a vascular complication of TRAS was suspected. Diagnostic arteriography was performed through a retrograde contralateral femoral artery puncture on 22nd day after transplantation, and it showed a 90% stenosis of transplant renal artery, and the TRAS occurred in the distal site of the anastomosis instead of the anastomosis (Fig. 1). We considered that the reconstructional renal artery branch stretched the trunk of renal artery, which resulted in the stenosis distal to the suture line. Two days later, after sufficient preparation renal artery angioplasty was undertaken through a retrograde ipsilateral femoral artery approach. As the stenosis might be high elastic due to the stretch, percutaneous transluminal stent implantation was performed. Because the artery branch situated on the stenosis, bare stent (6 × 14 mm, Express Vascular SD) had to be chosen to avoid effecting the flow of the artery branch. After the bare stent was successfully deployed, a second angiographic evaluation verified the effectiveness of the intervention was obvious. After the interventional treatment, the renal function and urine volume recovered, and his blood pressure was stably controlled to 121/80 mmHg with only two antihypertension (nifedipine and arotinolol). He was discharged on 28 day after transplantation with a creatinine of 108 μmol/L. Changes in clinical parameters over the 1-month treatment period is shown in Table 1. The patient’s renal function remains stable at clinical follow-up of 15 months.

**Table 1:** Clinical parameters changes over the 1-month treatment period

Date	Antihypertensive medications	Serum creatinine (μmol/L)	Urine output (ml/day)	Blood pressure in mmHg
Preoperative	Nifedipine 60 mg twice daily	1110	50	167/82
Postoperative day 1	intravenous Nicardipine 20 mg	768	4120	156/105
Postoperative day 3	Nicardipine 40 mg twice daily, spironolactone 20 mg twice daily, and furosemide 20 mg thrice daily	334	2040	152/103
Postoperative day 5	See above	238	1937	148/102
Postoperative day 7	See above	180	2265	153/104
Postoperative day 11	See above	151	3430	141/97
Postoperative day 14	Nicardipine 80 mg twice daily, spironolactone 20 mg twice daily, and furosemide 20 mg thrice daily	158	3540	170/103
Postoperative day 21	Nifedipine 60 mg twice daily, urapidil 30 mg twice daily, arotinolol 10 mg twice daily	194	1710	190/120
Postoperative day 22	Diagnostic arteriography			
Postoperative day 24	Percutaneous transluminal stent implantation			
Postoperative day 28	Nifedipine 30 mg once daily, arotinolol 10 mg once daily	108	2400	121/80

## Discussion and conclusions

TRAS is a relatively frequent, potentially curable vascular complication resulting in refractory hypertension and allograft dysfunction, and its incidence varies from 1% to 23%, which depends on the different definitions and diagnostic techniques [1]. At most centers arteriography will only be performed in patients with refractory hypertension or allograft dysfunction, so the true incidence of arterial stenosis posttransplantation may be underestimated.

TRAS usually occurs 3 months to 2 years after transplantation. It can present at different time and location depending on different causes. Several etiologies for explaining the development of TRAS have been proposed. The first one is atherosclerosis in the donor or recipient artery. Stenoses occurring later, ordinarily reflect atherosclerotic lesion either of the transplant renal artery or of the adjacent recipient iliac artery [3]. The second one is incorrect suture technique,, which causes localized stenosis confined to the suture line. Stenoses secondary to suture technique usually arise early after transplantation. The newest study, however, considered that the different types of arterial anastomoses were not proven to be a significant factor in relation to TRAS [4]. The third one is traumatic reasons which include damage to donor artery by traction, clamping, snaring or improper perfusion cannulation, and damage to the recipient iliac artery during transplantation. The other predisposing factors include increased cold ischemia time [5], cyclosporine toxicity [6], an immunologic cause [7], and cytomegalovirus infection.

The above-mentioned causes could not perfectly explain the TRAS in our case. Although the kinking of the renal artery occurred, it did not result from the redundant artery, but from the stretch of a superior polar artery branch. When the location of transplanted kidney changed from oblique to upright position in the right iliac fossa, the artery branch that became shorter after reconstruction by end-to-end anastomosis stretched the trunk of renal artery, which caused the kinking of the renal artery and then the stenosis occurred.

TRAS frequently presents with worsening or refractory hypertension and/or graft dysfunction. The most remarkable presentation in this case was refractory hypertension. Combined with the mild impaired renal function and reduced urine volume, TRAS was initially taken into consideration. Although a bruit was audible over the site of the transplanted kidney, it was of limited diagnostic value. Moreover, significant TRAS also can occur in the absence of an audible bruit [8].

Color Doppler ultrasonography, as an easily accessible and noninvasive procedure, is the first choice for routine screening procedure. The Color Doppler ultrasonography criteria for diagnosis are a PSV of higher than 200 cm/s in the renal artery, a RI of lower than 0.5, and a velocity gradient between stenotic and prestenotic segment of greater than 2:1 [9]. Spiral computed tomography (CT) clearly provides three-dimensional images of the vessels, however, its disadvantage is requiring contrast medium which may has nephrotoxicity. Diagnostic arteriography provides the definitive diagnosis of TRAS.

Once the diagnosis is confirmed, an appropriate and timely treatment is essential to preserve the allograft. Generally a reduction in the diameter of the renal artery greater than 50%, or any of the arteries leading up to it (proximal to the transplant renal artery) should be treated [10, 11]. Percutaneous intervention procedure is the preferred initial mode of therapy [12, 13]. The intervention procedure includes percutaneous transluminal angioplasty (PTA) and stent implantation. The former behaves well for stenosis that is short, linear, and relatively distal from the anastomosis [14]. Stent implantation is indicated when insufficient angioplasty (> 30% residual stenosis); stenosis within 3 months posttransplantation; flow-limiting dissection and high elastic recoil [15]. In this case the stenosis might be high elastic due to the stretch, so after arteriography we straightforwardly performed the stent implantation and second arteriography proved a perfect effectiveness. For avoiding side branch artery occlusion, a bare metal stent was deployed across the stenosis, and obvious patency of the artery branch was showed. Kolli considers that stent implantation is not applied to stenosis due to a kink in the transplant renal artery, since the kink may propagate to the distal of the stent [16]. In our case the stent implantation did not result in propagation of the kink. A guideline approved by the Society of Cardiovascular and Interventional Radiology defined technical success of percutaneous intervention as < 20% residual stenosis with no signs of flow-limiting dissection, and clinical success was defined as complete relief or substantial improvement in presenting symptoms [17]. Studies published over the last decade reported that the interventional procedure has high technical success rate (range from 92.3%–100%) and clinical success rates (range from 75%–86%) [18–20]. After stenting procedure restenosis rates of 0%–22% in individual series and 9.6% on pooled analysis of several studies (*n* = 294) were reported [21]. Until now more than one year after intervention our case still presents with a stable renal function with the creatinine of 91 umol/L and blood pressure, which indicate the patency of the renal artery. The reported rate of complications such as hematoma, aneurysm, arterial rupture, embolization, infarctis usually 0% to 10% [17]. Fortunately, our patient has a calm recover without any complications.

In conclusion, we present a special patient with TRAS caused by stretch of an artery branch, a very rare reason, and he was successfully cured by the interventional placement of stent and has a stable renal function on follow-up. We expect that introducing this case could provide some experience to clinical doctors when they deal with similar patients later.

## Abbreviations

TRAS: Transplant renal artery stenosis
RI: Resistance index
PSV: Peak systolic velocity
PTA: Percutaneous transluminal angioplasty
